# Quality of public health insurance and individuals’ consumption structure upgrades: evidence from China

**DOI:** 10.1186/s13561-021-00343-x

**Published:** 2021-12-03

**Authors:** Pengfei Zhang, Jinghua Gao

**Affiliations:** 1 School of Labor and Human Resources, Renmin University of China, No 59, Zhongguancun Street, Beijing, 100872 China; 2 Centre for Social Investment (CSI), Heidelberg University, Bergheimer Str. 58, 69115 Heidelberg, Germany

**Keywords:** Public health insurance, Quality, Consumption structure upgrade

## Abstract

**Background:**

The aim of this study was to investigate the relationship between the quality of public health insurance and individuals’ consumption structure upgrades in China.

**Methods:**

This study was conducted using data from a population of 6430 Chinese individuals aged 18 to 70 y from the 2017 Chinese Social Survey. We used multiple linear regression models and the two-stage least-squares model to explore the impact and heterogeneity of the quality of public health insurance on individuals’ consumption structure upgrades. Furthermore, we performed structural equation modelling to clarify the mediation effects of the impact.

**Results:**

The quality of public health insurance was significantly correlated with individuals’ consumption structure upgrades (β = 0.368, SD = 0.084), and the impact of the quality of public health insurance on individuals’ hedonic consumption in urban regions was significantly higher than that in rural regions (β = 0.499, SD = 0.218). Furthermore, the quality of public health insurance could promote upgrades to individuals’ consumption structure by reducing the burden of medical expenses and stabilizing or increasing individuals’ expectations regarding the future economic level.

**Conclusion:**

The results indicated that developing countries should implement additional measures to enhance the quality of public health insurance, which would not only help protect the health of individuals but also stimulate individuals’ consumption to achieve rapid economic growth.

## Background

In the past, the economic development of China, the second-largest economy in the world, relied mainly on investment. However, the traditional approach of using investment to drive economic development has become increasingly unsuitable for China’s actual environment. In the case of relatively stable consumption, investment-driven economic development introduces surplus supply and even excess capacity [1, 2]. To maintain the investment-driven economic growth over a long period, excess capacity can only be alleviated by squeezing exports, which aggravates the pressure on exports [3, 4]. Although investment can drive consumption to a certain extent, the elasticity coefficient of investment-driven consumption in China is relatively low compared to that in other developed countries [5, 6]. This phenomenon occurs because in the process of China’s investment-driven economic development, the growth rate of consumption is significantly lower than that of investment and considerably lower than the accumulation rate of the excess capacity. Therefore, China can achieve high-quality economic development only by transforming the investment-driven economic development mode into a consumption-driven economic development mode, thereby promoting individuals’ consumption, driving economic growth with consumption, and stimulating effective supply with consumption [7–10]. In recent years, with the gradual transformation of China’s economic development mode, continuous optimization and transformation of the country’s economic structure, and adjustment of its economic growth momentum, the contribution of consumption to the gross domestic product (GDP) has gradually increased. However, compared with that in developed countries, the proportion of domestic consumption in GDP is still low, with much room for improvement. Therefore, promoting the upgrading of the individuals’ consumption structure is the key to achieving high-quality economic development in China.

Individuals’ consumption structure upgrades are closely related to health. Rapid health changes force individuals to increase their necessary health consumption. Considering the total consumption, an increase in the health consumption is expected to lead to a decrease in other types of consumption, especially hedonic consumption [11–14]. Moreover, health consumption is different from other types of consumption. In particular, health consumption demand is rigid in nature, and individuals must correspondingly consume to fulfil their medical demand [15–17]. Moreover, health consumption involves significant uncertainty owing to the uncertainty of disease risks [18–20]. In other words, in cases involving disease risks, individuals’ health consumption is unpredictable, and critical diseases may cause individuals to fall into poverty [21–23]. In addition, Chinese individuals have a higher willingness to save, primarily to prevent the excessive impact of health decline on their own consumption [24, 25].

The quality of public health insurance reflects the individual’s evaluation of the public health insurance. When individuals consider the public health insurance sponsored by the government to be satisfactory, the quality of public health insurance is high. In cases involving disease risks, high-quality public health insurance can provide the individual with economic compensation, reduce the burden of medical expenses borne by individuals, and allow the funds that would be used for health consumption to be used for other types of consumption. Moreover, high-quality public health insurance can ease an individual’s concerns regarding uncertain disease risks, reduce individual deposits, and promote individuals’ consumption.

The current research on the impact of the quality of public health insurance on individuals’ consumption structure upgrades remains limited. Furthermore, the mechanisms that influence the quality of public health insurance and individuals’ consumption structure upgrades have not been explored. In this study, we considered three relevant aspects. First, we examined the impact of the quality of public health insurance on individuals’ consumption structure upgrades in China and provide new perspectives on ways to improve individuals’ consumption in China. Second, we explored the heterogeneity of this impact in urban and rural areas. The public health insurance systems in urban and rural areas are considerably different, which may lead to heterogeneity in the impact of the quality of public health insurance on individuals’ consumption structure upgrades. Third, we examined the mechanism of the impact of the quality of public health insurance on individuals’ consumption structure upgrades.

### Burden of medical expenses

The consumption capacity is restricted not only by income but also by the consumption structure. In the case of a certain income, the individual’s consumption capacity remains relatively stable. When an individual exhibits excessive consumption in one field, the consumption in other fields is expected to decrease. In other words, for a certain consumption ability, a crowding out effect exists among individual consumption types. Medical expenses are a part of basic consumption in an individual’s life. When diseases occur, individuals incur certain medical expenses in the process of fulfilling their medical demand to maintain their health. These medical expenses and other types of consumptions constitute the individual’s consumption structure. If the medical burden faced by the individual is excessively high, the individual tends to expend a larger part of his or her consumption ability on health-related expenses to fulfil the medical demand and is forced to spend less in other fields. Therefore, the excessive medical burden exerts a crowding out effect on other types of individual consumptions, leading to an imbalance in the individual consumption structure. Specifically, if individuals expend excessive resources to fulfil their medical demand, the hedonistic consumption is reduced, leading to degraded individual consumption. The purpose of enhancing the quality of public health insurance is to more effectively protect the health of individuals and eliminate individuals’ concerns regarding disease risks. The medical expenses expected to be borne by individuals can be shared among all insured people by fully exploiting the risk sharing mechanism. In this scenario, the basic consumption amount that individuals must spend to maintain their own health is considerably reduced, and subsequently, a greater percentage of the consumption ability can be used for their personal development and interests.

### Individuals’ expectations regarding the future economic level

The unpredictability of the future economic level is a key reason for the downturn in individual consumption. Due to the unpredictability of the individuals’ future economic level, to gain more control over the future economic level, individuals tends to save more money and consume less. Disease risk is a type of unpredictable risk that adversely influences an individual’s personal economic level. When unexpected disease risk occurs, individuals need to bear certain medical expenses to realize their own medical demand, which reduces the disposable income of individuals and deteriorates their economic levels. Moreover, individuals bear certain time costs in the process of realizing their medical demand, which may reduce their normal employment income. Disease risks not only deteriorate the disposable income and normal work income of individuals but may also incur the cost of care, which reduces the economic level of the individuals to a certain extent. Therefore, disease risks, as an unpredictable factor, increase the unpredictability of the individual’s future economic level, forcing the individual to transfer part of his or her funds originally used for consumption to savings to reduce the unpredictability of the future economic level. Enhancing the quality of public health insurance can help eliminate individuals’ concerns regarding disease risks. Even after disease risks occur, economic compensation can be provided to reduce the impact of the disease risks on an individual’s economic level to a certain extent, which can enhance the individual’s expectations of the future economic level and promote individual consumption instead of saving.

## Methods

### Data

The data for the analysis were extracted from the Chinese Social Survey (CSS). The CSS is a nationally representative survey sponsored by the Institute of Sociology, Chinese Academy of Social Sciences. The CSS was initiated in 2005 to obtain data regarding China’s social changes during the transitional period by surveying the employment, family, social life, and social attitudes of the national population, thereby providing abundant data for social science research and inform government decision-making. Based on probability sampling and household visits, the CSS is conducted every two years. The survey area of the CSS covers 31 provinces, 151 county-level districts, and 604 villages/neighbourhood committees. The age of the survey respondents ranges from 18 to 70 y.

The CSS questionnaire is divided into the following three parts: basic module, replacement module and hotspot module. The content of the basic module is fixed, including basic personal information, labour and employment, family structure, and family economic status. The content of the replacement module, which mainly includes social class, social security, leisure consumption, and social value information, is constantly adjusted. The content of the hotspot module is related to issues that society focuses on, such as social interest relations, livelihood issues, and urbanization. In 2016, the Chinese government announced its aim to improve the quality of public health insurance, and the CSS introduced this timely social issue into the questionnaire in 2017. Therefore, the data considered in this work are cross-sectional data from the 2017 CSS.

In 2017, the CSS began to investigate the Chinese population’s evaluation of public health insurance to reflect the quality of China’s public health insurance. The quality of public health insurance was reflected by the response to the question, “What do you think of the public health insurance provided by the Chinese government?”. The respondents’ answers were divided into the following four categories: very good, relatively good, not good, and very bad. Based on the respondents’ answers, we integrated the “very good” and “relatively good” responses into the “satisfactory evaluation” standard and assigned it a value of “1″, which reflected the opinion that China’s public health insurance is of high quality. The “not good” and “very bad” responses were combined into the “unsatisfactory evaluation” standard and assigned a value of “0″, which reflected the opinion that China’s public health insurance is of low quality.

Consumption can be divided into the following types: survival, development and hedonic consumptions [26, 27]. Survival consumption represents the necessary consumption for individuals to survive and pertains to food, clothing, housing and transportation. Development consumption indicates the consumption of goods and services to meet individuals’ development needs. Hedonic consumption is the consumption of leisure, entertainment, tourism, and cultural goods and services by individuals. Individuals’ consumption structure upgrades focus on the change from survival and development consumptions to hedonic consumption [28, 29]. In the process of promoting the upgrading of the individuals’ consumption structure, the Chinese government emphasized the enhancement of the proportion of hedonic consumption in the total consumption [30]. The basic module of the 2017 CSS contains detailed information regarding the individual consumption, including the total, cultural, tourism, leisure and entertainment consumptions. Based on this information, we can calculate the proportion of hedonic consumption in the total consumption, which can indicate the change in individuals’ consumption structure.

Because investigating whether the quality of public health insurance can promote the upgrading of the individuals’ consumption structure by reducing the burden of medical expenses and stabilizing or increasing individuals’ expectations regarding the future economic level, we must evaluate the burden of individuals’ medical expenses and expectations regarding the future economic level. In the 2017 CSS questionnaire, the burden related to individuals’ medical expenses was reflected by the response to the question “Which of the following life problems have you encountered?”. One of the options was “Medical expenses are too great to bear”. If a respondent chose this option, it meant that his or her burden from medical expenses was significant, and a value of “1″ was assigned. If a respondent did not choose this option, it meant that his or her burden from medical expenses was not significant, and a value of “0″ was assigned. In the 2017 CSS questionnaire, individuals’ expectations of the future economic level were reflected by the following two questions, “What do you think is your current economic status level?” and “What do you think your economic status level will be in the next 5 years?”. The respondents’ answers to the two questions were divided into five equally weighted categories: upper, middle upper, middle, middle lower and lower. We compared the respondents’ answers to these two questions. If the respondent’s answer to the latter question was a category not lower than that of the former question, it meant that the individual’s expectation regarding the future economic level was stable or upward moving, and a value of “1” was assigned. Otherwise, the individual’s expectation regarding the future economic level was downward moving, and a value of “0” was assigned.

Individual characteristics such as gender (1 = male, 0 = female), age, marital status (1 = married, 0 = unmarried), years of education (illiteracy = 0, primary school = 6, junior middle school = 9, high school = 12, university = 16, university above = 19), income, political status (1 = party member, 0 = non-party member), Internet access (1 = yes, 0 = no), occupational status (1 = works, 0 = does not work), household size, and family relationship satisfaction were also considered in the regression analysis.

Table 1 reports the descriptive statistics of the variables. In terms of individuals’ consumption structure upgrades, the mean hedonic consumption in the total consumption of the entire population was relatively low in China (1.619%). The proportion of hedonic consumption in the urban population (2.360%) was higher than that in the rural population (1.416%). Most respondents (70.6%) considered the public health insurance to be satisfactory. Moreover, the proportion of the rural population that considered the public health insurance to be satisfactory (71.3%) was higher than that of the urban population (68.2%), indicating that the rural population’s satisfaction with public health insurance gradually increased since the Chinese government began to emphasize the enhancement of the quality of public health insurance in 2016.

**Table 1:** Descriptive statistics of variables

Sample	All Population		Urban Population		Rural Population	
	Mean/(SD)	N	Mean/(SD)	N	Mean/(SD)	N
	(1)	(2)	(3)	(4)	(5)	(6)
Individuals’ consumption structure upgrades (%)	1.619	6430	2.360	1384	1.416	5046
	(3.434)		(3.934)		(3.255)	
Quality of public health insurance	0.706	6430	0.682	1384	0.713	5046
	(0.456)		(0.466)		(0.452)	
Gender	0.459	6430	0.436	1384	0.465	5046
	(0.498)		(0.496)		(0.499)	
Age	45.144	6430	39.913	1384	46.578	5046
	(13.922)		(13.752)		(13.623)	
Marriage	0.882	6430	0.831	1384	0.895	5046
	(0.323)		(0.375)		(0.306)	
Years of education	9.135	6430	10.463	1384	8.772	5046
	(4.248)		(4.051)		(4.228)	
Political status	0.098	6430	0.100	1384	0.098	5046
	(0.298)		(0.301)		(0.297)	
Internet access	0.438	6430	0.589	1384	0.396	5046
	(0.496)		(0.492)		(0.489)	
Whether works or not	0.650	6430	0.611	1384	0.661	5046
	(0.477)		(0.488)		(0.473)	
Income (logarithm)	7.934	6430	7.974	1384	7.923	5046
	(3.840)		(4.234)		(3.725)	
The household size	4.373	6430	4.013	1384	4.471	5046
	(1.831)		(1.548)		(1.889)	
Family relationship satisfaction	8.446	6430	8.521	1384	8.426	5046
	(1.923)		(1.851)		(1.942)	

Notes: Monetary units are in 2017 Chinese yuan. Standard deviations are in parentheses

### Empirical strategy

The research object was the effect of the quality of public health insurance on individuals’ consumption structure upgrades, defined as

1 $$ {Upgrade}_i={\beta}_0+{\beta}_1{quality}_i+{X}_i\upgamma +{\varepsilon}_i $$

where i denotes an individual; *Upgrade*_*i*_ indicates an individual’s consumption structure upgrade; and *quality*_*i*_ indicates the quality of public health insurance. *X*_*i*_ denotes individual i’s characteristics, household characteristics, and district fixed effects. The parameter of interest was *β*_1_.

Furthermore, we examined the heterogeneity in the influence of the quality of public health insurance on the upgrade in individuals’ consumption structure in urban and rural regions. Based on Eq. (1), we established the following equation:

2 $$ {Upgrade}_i={\alpha}_0+{\alpha}_1 qualit{\mathrm{y}}_i+{\alpha}_2 region+{\alpha}_3{quality}_i\times region+{X}_i\upgamma +{\varepsilon}_i $$

where *region* is a dummy variable for the region. For individuals belonging to urban and rural regions, we assigned this dummy variable a value of “1” and “0”, respectively. The parameter of interest was *α*_3_.

The public health insurance quality may be endogenous, as the factors affecting individuals’ consumption structure upgrades are highly complex, owing to which certain important variables may be neglected. To avoid this neglect, by using the multiple linear regression method, we adopted the two-stage least squares method to evaluate the robustness. We selected the following two instrumental variables: an individual’s trust in the local government, and the fairness of the public health insurance. In general, the individuals’ trust in the local government is related to the promotion and popularization of public health insurance policies and measures in the local area [31, 32]. High trust in the local government can lead to a smooth implementation of public health insurance policies and measures, which can ensure that public health insurance achieves its full effect. Therefore, by enhancing trust in the local government, the quality of the public health insurance can be enhanced. The fairness of public health insurance reflects whether there exist equal opportunities for individuals to participate in public health insurance and access medical services, which is closely related to the realization of individuals’ medical demand [33, 34]. Therefore, fair public health insurance is a sufficient condition for ensuring high-quality public health insurance. Notably, there is no evidence to show that an individual’s trust in the local government and fairness of public health insurance are directly related to an individual’s consumption structure upgrade. Therefore, according to experience, the two considered instrumental variables are related to the quality of public health insurance and not related to the random disturbance term. In addition, we examined whether the considered instrumental variables have such properties based on subsequent tests in the two-stage least squares method. In the 2017 CSS questionnaire, trust in the local government was reflected by the response to the question, “Do you trust your local government?”. The respondents’ answers were divided into the following four categories: no trust, lack of trust, high trust and full trust. Based on the respondents’ answers, we integrated the “high trust” and “full trust” responses into the “trust” standard, which was assigned a value of “1” and reflected a high level of trust in the local government. The “no trust” and “lack of trust” responses were combined into the “distrust” standard and assigned a value of “0”, which reflected low trust in the local government. In the 2017 CSS questionnaire, the fairness of public health insurance was reflected by the response to the question “Do you think the public health insurance sponsored by the government is fair?”. The respondents’ answers were divided into four categories: very unfair, not very fair, relatively fair and very fair. Based on the respondents’ answers, we integrated the “relatively fair” and “very fair” responses into the “fair” standard, which was assigned a value of “1” and reflected the opinion that the public health insurance was fair. The “very unfair” and “not very fair” responses were combined into the “unfair” standard and assigned a value of “0”, which reflected the opinion that public health insurance was not entirely fair.

Finally, to clarify the impact mechanism of the quality of public health insurance on individuals’ consumption structure upgrades, we used the structural equation modelling (SEM) method to study the impact mechanism and calculated the magnitude of the mediation effect.

## Results

### Impact of the quality of public health insurance on individuals’ consumption structure upgrades

Panel A of Table 2 reports the impact of the public health insurance quality on upgrades to individuals’ consumption structure. The results show that the quality of public health insurance significantly affects individuals’ hedonic consumption (β = 0.368, SD = 0.084), which means that the quality of public health insurance can promote upgrades to individuals’ consumption structure. Panel C of Table 2 indicates the impact of the quality of public health insurance on individuals’ consumption structure upgrades by clustering based on the household size. The data show that the quality of public health insurance significantly affects individuals’ hedonic consumption. The hedonistic consumption of individuals with satisfactory evaluations of public health insurance is 0.368% higher. This regression coefficient seems relatively low, but it considerably influences the economic development. In 2017, 6.02% of the GDP pertained to individuals’ hedonic consumption [35]. By combining this number and regression coefficient, the enhancement in the quality of public health insurance corresponds to 1.37% (0.368%/1.619% × 6.02%) of the GDP for China’s economic development. If the Chinese government improves the quality of public health insurance to satisfy all individuals, China’s GDP can increase by 0.57% (1.37%/0.706 × 0.294). Therefore, enhancing the quality of public health insurance can significantly promote upgrades to individuals’ consumption structure and influence China’s economic development.

**Table 2:** Baseline regression results

Variables	A	B	C	D
Quality of Public Health Insurance	0.368***	0.266***	0.368***	0.266***
	(0.084)	(0.092)	(0.076)	(0.038)
Region		0.079		0.079
		(0.174)		(0.294)
Quality of Public Health Insurance × Region		0.499**		0.499**
		(0.218)		(0.237)
Observations	6430	6430	6430	6430
R-squared	0.140	0.143	0.140	0.143

Note: The dependent variables of Panel A, Panel B, Panel C and Panel D are individuals’ consumption structure upgrades. All Panel A, Panel B, Panel C and Panel D include gender, age, marriage, years of education, income, political status, Internet access, whether the individual works or not, household size, family relationship satisfaction, and province effects. Robust standard errors are reported in parentheses. *** *p* < 0.01, ** *p* < 0.05, * *p* < 0.1

### Heterogeneity in the influence of the quality of public health insurance on individuals’ consumption structure upgrades in urban and rural regions

Panel B of Table 2 reports the heterogeneity in the influence of the quality of public health insurance on individuals’ consumption structure upgrades in urban and rural regions. The impact of the quality of public health insurance on individuals’ hedonic consumption in urban regions is significantly higher than that in rural regions (β = 0.499, SD = 0.218). Panel D of Table 2 summarizes this heterogeneity by clustering based on household size. The data show that individuals’ hedonic consumption in urban regions is significantly higher than that in rural regions. This heterogeneity can be attributed to the difference in the levels of public health insurance benefits in urban and rural regions. Although China has achieved universal health coverage through public health insurance, certain differences remain in terms of the levels of public health insurance benefits within the system. For example, the basic public health insurance for urban employees (BUE), basic public health insurance for urban unemployed residents (BUR) and new rural cooperative public health system (NRC) involve certain differences in the benefit levels. The BUE has the highest level of benefits. When faced with a disease, the reimbursement proportion of medical expenses is the highest for urban employees. The BUR has a relatively high level of benefits, and the reimbursement proportion of medical expenses is relatively high for unemployed urban residents. The NRC has the lowest level of benefits, and the reimbursement proportion of medical expenses is the lowest for the rural population. Due to the higher reimbursement proportion of medical expenses for the urban population, the urban population’s burden associated with medical expenses is lower than that of the rural population, and the urban population’s expectations regarding the future economic level are higher than those of rural residents; thus, the urban population engages in more hedonic consumption than the rural population.

### Robustness test

We adopted the two-stage least squares method for the robustness test. Panel A of Table 3 reports the robustness test results for the impact of the quality of public health insurance on individuals’ consumption structure upgrades. The Kleibergen–Paap rk LM statistic and Hansen J statistic indicate that the considered instrumental variables are highly correlated with the quality of public health insurance and independent of the unobservable error process, which shows that the instrumental variables are valid. Panel A shows that the quality of public health insurance can significantly enhance individuals’ hedonic consumption (β = 1.016, SD = 0.317), which means that the quality of public health insurance can promote upgrades to individuals’ consumption structure.

**Table 3:** Regression results of the two-stage least square

Variables	A	B
Quality of Public Health Insurance	1.016***	0.696**
	(0.317)	(0.341)
Region		−0.905
		(0.590)
Quality of Public Health Insurance×Region		1.958**
		(0.857)
Observations	6430	6430
Kleibergen-Paap rk LM statistic	401.540	91.955
	[0.000]	[0.000]
Hansen J statistic	2.622	2.388
	[0.105]	[0.303]

Note: The dependent variables of Panel A and Panel B are Individuals’ consumption structure upgrades. Panel A and Panel B both include gender, age, marriage, years of education, income, political status, Internet access, whether the individual works or not, household size, family relationship satisfaction, and province effects. Robust standard errors are reported in parentheses, and *p*-values are reported in square brackets. To test whether the instrumental variables are weakly correlated with the endogenous variables, we use the Kleibergen-Paap rk LM statistic, and rejection of the null hypothesis indicates that the instruments have strong correlation with endogenous variables [36]. To test whether the instrumental variables are independent from the unobservable error process, we use the Hansen J statistic, and non-rejection of the null hypothesis indicates that the instruments satisfy the orthogonally condition [37]. *** *p* < 0.01, ** *p* < 0.05, * *p* < 0.1

Panel B of Table 3 summarizes the results of the robustness test to determine whether the influence of public health insurance quality on individuals’ consumption structure upgrades in urban and rural regions is heterogeneous. The Kleibergen–Paap rk LM statistic and the Hansen J statistic still indicate that the considered instrumental variables are valid. After resolving the problem of endogeneity in the quality of public health insurance, it was noted that the impact of the quality of public health insurance on individuals’ hedonic consumption in urban regions was still significantly higher than that in rural regions (β = 1.958, SD = 0.857).

### Possible mechanisms

We used the SEM method to investigate the possible mechanisms through which the quality of public health insurance affected the individuals’ consumption structure upgrades.

The burden from medical expenses is a key mediating factor for the influence of the quality of public health insurance on individuals’ consumption structure upgrades. The individual consumption capacity is restricted not only by income but also by its consumption structure. In the case of a set income, the individual’s consumption capacity remains relatively stable. When the individual consumes excessively in one field, the consumption in other fields decreases. In other words, for a specific consumption capacity, a crowding out effect occurs among the different consumption types [38, 39]. Medical expenses are a type of consumption in individuals’ lives. In the case of disease occurrence, individuals must bear certain medical expenses in the process of realizing their medical demand to maintain their health. Therefore, medical expenses together with other individual consumption types form the individual consumption structure. If an individual experiences a heavy burden from medical expenses, the individual must expend a larger part of his or her consumption ability to fulfil his or her own medical demands and is forced to spend less in other fields of life, such as hedonic consumption. Therefore, the heavy burden from medical expenses has a crowding out effect on the individual’s other types of consumption, leading to an imbalance in the individual’s consumption structure [40, 41]. In other words, if an individual spends excessively to maintain his or her basic life, the individual’s hedonic consumption is reduced, leading to a degraded consumption structure. The purpose of enhancing the quality of public health insurance is to more effectively protect the health of individuals and eliminate the concerns of individuals regarding disease risks. The “law of large numbers” and principle of risk sharing can be used to share the burden of medical expenses that originally need to be borne by an individual to the entire insured population. This framework not only satisfies an individual’s medical demand but also considerably reduces the individual’s burden associated with medical expenses [42, 43]. In this scenario, the basic consumption of individuals to maintain their own health is considerably reduced, and more of the consumption ability can be used for hedonic purchases. Therefore, enhancing the quality of public health insurance can alleviate the restriction that basic medical consumption imposes on individuals’ hedonic consumption by reducing the burden of medical expenses, which can promote the upgrading of individuals’ consumption structure.

An individual’s expectation regarding the future economic level is a key factor mediating the influence of the quality of public health insurance on individuals’ consumption structure upgrades. An individual’s uncertain future economic level is a notable reason for reduced consumption [44, 45]. Due to the uncertain future economic level, individuals tend to save more funds and consume less to strengthen their control over their future economic level [24]. Notably, disease risks are unexpected and adversely impact an individual’s future economic level. When unexpected disease risks occur, individuals must bear medical expenses to meet their own medical demand, which directly reduces their disposable incomes. Moreover, individuals may have to bear the costs of time in the process of realizing their own medical demand, which may reduce their normal income. In addition, for populations faced with more critical diseases, the disposable income and normal income from work are reduced, and care costs may be incurred, which also reduces the economic level of the individuals to a certain extent. Therefore, disease risks, as an unexpected factor, increase an individual’s unexpected future economic level, forcing the individual to transfer a part of his or her funds originally used for consumption to savings [46, 47]. The quality of public health insurance can be enhanced to eliminate the residents’ concerns regarding disease risks. Even after the occurrence of disease risks, economic compensation may be provided to reduce the impact of disease risks on an individual’s economic level to a certain extent. Therefore, enhancing the quality of public health insurance can reduce an individual’s concerns regarding unexpected disease risks, improve the individual’s expectations regarding the future economic level, and encourage the individual to spend more money on hedonic consumption rather than saving.

**Fig. 1:**
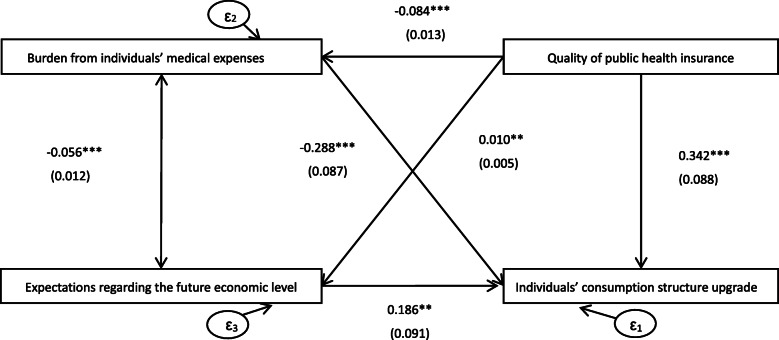
Analysis of Mechanisms for Quality of Public Health Insurance Affecting Individuals’ Consumption Structure Upgrades. Note: All specifications include gender, age, marriage, years of education, income, political status, Internet access, whether the individual works or not, the household size, family relationship satisfaction, and province effects. RSMA< 0.01, SRMR< 0.01, CFI > 0.95, TFI > 0.95. Standard errors are reported in parentheses. *** *p* < 0.01, ** *p* < 0.05, * *p* < 0.1

Figure 1 shows the SEM regression results. The results exhibit a satisfactory fit to the data (RSMA< 0.01, SRMR< 0.01, CFI > 0.95, TFI > 0.95). The results show that the quality of public health insurance (β = 0.342, SD = 0.088) can promote individuals’ consumption structure upgrades, which confirms the robustness of the estimation of this study. Moreover, the quality of public health insurance negatively influences the burden of medical expenses (β = − 0.084, SD = 0.013) and positively influences individuals’ expectations of the future economic level (β = 0.010, SD = 0.005). Moreover, the burden of medical expenses (β = − 0.288, SD = 0.087) negatively impacts individuals’ consumption structure upgrades, and individuals’ expectations regarding the future economic level (β = 0.186 SD = 0.091) positively influences individuals’ consumption structure upgrades. In addition, the burden of medical expenses and individuals’ expectations regarding the future economic level exhibit a two-way negative relationship (β = − 0.056, SD = 0.012).

Table 4 reports the decomposition of the effects of the quality of public health insurance on individuals’ consumption structure upgrades. The results show that the indirect effect of the quality of public health insurance on individuals’ consumption structure upgrades through the burden of medical expenses is 0.024, accounting for 7.69% of the total effect; the indirect effect of the quality of public health insurance on individuals’ consumption structure upgrades through individuals’ expectations regarding the future economic level is 0.002, accounting for 1.06% of the total effect. In summary, the burden of medical expenses and individuals’ expectations regarding the future economic level play notable mediation roles, with the burden of medical expenses having a higher mediation effect. The total effect of the quality of public health insurance on individuals’ consumption structure upgrades is 0.368, and the mediation effect through the burden of medical expenses and individuals’ expectations regarding the future economic level is 0.026, accounting for 7.07% of the total effect. This finding indicates that the two mediation mechanisms can accurately explain part of how the quality of public health insurance affects individuals’ consumption structure upgrades.

**Table 4:** Decomposition of the effects of quality of public health insurance on the individuals’ consumption structure upgrades

Variables	Direct effects	Indirect effects	Total effects	Percentage of indirect effects
Burden from individuals’ medical expenses	0.288	0.024	0.312	7.69%
Individuals’ expectations regarding the future economic level	0.186	0.002	0.188	1.06%
Individuals’ consumption structure upgrades	0.342	0.026	0.368	7.07%

Note: The indirect mediation effects are the products of the corresponding two mechanisms coefficients, respectively; the total mediation effects are the sums of direct effects and indirect effects, respectively

## Discussion

The quality of public health insurance can significantly increase individuals’ hedonic consumption (β = 0.368, SD = 0.084), which shows that the quality of public health insurance significantly affects individuals’ consumption structure upgrades. In 2017, 6.02% of the GDP corresponded to the hedonic consumption [35]. By combining this number with the regression coefficient, we estimated the contribution of the quality of public health insurance to China’s GDP. The quality of public health insurance contributes 1.37% of China’s GDP, corresponding to a notable impact.

Next, we investigated the heterogeneity in the influence of the quality of public health insurance on individuals’ consumption structure upgrades and noted a significant difference between urban and rural areas in terms of the impact of the quality of public health insurance on individuals’ consumption structure upgrades. The impact of the quality of public health insurance on individuals’ hedonic consumption in urban regions is significantly higher than that in rural regions (β = 0.499, SD = 0.218). The Chinese government has implemented different public health insurance systems in urban and rural areas, which is the main reason for the heterogeneity of the impact.

We tested the robustness of the abovementioned two main findings. The test results supported our findings and highlighted that our findings are robust.

Finally, we explored the mechanism by which the quality of public health insurance affects individuals’ consumption structure upgrades and calculated the magnitude of the mediation effect. The quality of public health insurance can promote individuals’ consumption structure upgrades by reducing the burden of medical expenses and stabilizing or increasing individuals’ expectations regarding the future economic level. The mediation effect of reducing the burden of medical expenses is stronger than that of stabilizing or increasing individuals’ expectations regarding the future economic level.

When promoting economic development, investment and consumption occupy the leading positions. In the past, China’s main approach was to expand the proportion of investment in economic growth. Although this method helped maintained the growth of China’s economy, the growth rate has gradually decreased in recent years. Moreover, the excessive emphasis on investment has led to almost no significant changes in China’s consumption structure in recent decades. Although excess supply may promote economic growth by serving the external market to a certain extent, the marginal utility of this approach in China is decreasing. Over the past decade, although China has maintained a large trade surplus, the economic growth rate has declined yearly. Therefore, Chinese economists have gradually realized that promoting consumption is a valuable approach to solve the problem of slow economic growth in China. Moreover, by promoting consumption upgrading, the investment and consumption proportions in economic growth can be optimized, and an excessive proportion of investment in economic growth can be avoided, thereby contributing to the realization of high-quality economic development.

This study contributes to the literature by providing new evidence of the impact of public health insurance on individuals’ consumption. Our finding that public health insurance promotes individuals’ consumption is similar to previous findings regarding the consumption effect of public health insurance sponsored by the government [13, 14, 48–50]. However, the subject of the existing research was the impact of public health insurance on individuals’ consumption, with a focus on comparing the state before the implementation of public health insurance and changes in individuals’ consumption after the implementation. The key findings of the existing research advocated for the government to expand the coverage of public health insurance. The difference between this study and the existing studies is that this study focuses on the impact of the quality of public health insurance on consumption, emphasizing the quality of public health insurance, rather than the public health insurance system. Moreover, the conclusions derived in this study provide a reference direction for the future development of public health insurance, which is to encourage individuals’ consumption and promote economic development by continuously improving the quality of public health insurance, rather than by simply improving the coverage of public health insurance.

This study also contributes to the literature by exploring the heterogeneity in the influence of the quality of public health insurance on individuals’ consumption structure upgrades and the associated potential mechanisms. Based on abundant survey data, we examined whether the impact in urban and rural regions is heterogeneous and whether the burden of medical expenses and individuals’ expectations regarding the future economic level are mediation variables. The systems of public health insurance in urban and rural areas in China are different. The urban population enjoys more benefits from public health insurance than the rural population, and thus, the quality of public health insurance in rural regions is lower than that in urban regions, resulting in heterogeneous impacts. Our findings confirm the inequity of China’s public health insurance due to the urban–rural division, which validates the findings of previous studies [51–54]. In addition, our findings not only confirm that public health insurance is closely related to the burden that individuals face from medical expenses, consistent with the findings of previous studies [55–58], but also confirm that public health insurance is closely related to individuals’ expectations regarding the future economic level, which expands the existing literature [59–62].

Nevertheless, this study has certain limitations. First, we considered individuals’ evaluation of public health insurance to reflect the quality of public health insurance. This measurement method is consistent with the current development of public health insurance in China. For other developing countries, the measurement of the quality of public health insurance must be further assessed. Second, we clarified certain possible mechanisms by which the quality of public health insurance may affect individuals’ consumption structure upgrades; however, other possible mechanisms must also be investigated.

## Conclusion

We identify the relationship between the quality of public health insurance and individuals’ consumption structure upgrades. Our results indicate that the quality of public health insurance can promote individuals’ consumption structure upgrades. Moreover, the impact of the quality of public health insurance on individuals’ hedonistic consumption in urban regions is significantly higher than that in rural regions. In addition, the quality of public health insurance can promote individuals’ consumption structure upgrades by reducing the burden of medical expenses and stabilizing or increasing individuals’ expectations regarding the future economic level. Our analysis can explain why the burden of medical expenses and individuals’ expectations regarding the future economic level may be important mechanisms through which the quality of public health insurance affects individuals’ consumption structure upgrades and clarify the reasons for the differences between urban and rural areas in terms of the impact of the quality of public health insurance on individuals’ consumption structure upgrades.

In contrast to the existing studies, we emphasize the importance of the quality of public health insurance, rather than the importance of enhancing the coverage of public health insurance. With economic development, an increasing number of developing countries have established public health insurance systems and are committed to achieving universal health coverage. This study indicates that developing countries should introduce additional measures to improve the quality of public health insurance—not to only protect the health of individuals but also to stimulate individuals’ consumption and achieve rapid economic growth.

## Data Availability

The data that support the findings of this study are available from the Chinese Social Survey (http://css.cssn.cn/css_sy/) but restrictions apply to the availability of these data, which were used under license for the current study, and so are not publicly available. Data are however available from the authors upon reasonable request and with permission of the Chinese Social Survey.

## Abbreviations

GDP: Gross domestic product
BUE: Basic public health insurance for urban employees
NRC: New rural cooperative public health system
BUR: Basic public health insurance for urban unemployed residents
CSS: Chinese social survey
SEM: Structural equation modelling
